# Impact of Different Surface Ligands on the Optical Properties of PbS Quantum Dot Solids

**DOI:** 10.3390/ma8041858

**Published:** 2015-04-21

**Authors:** Fan Xu, Luis Felipe Gerlein, Xin Ma, Chelsea R. Haughn, Matthew F. Doty, Sylvain G. Cloutier

**Affiliations:** 1 Alphasense, Inc., 510 Philadelphia Pike, Wilmington, DE 19809, USA; E-Mail: xufan@udel.edu; 2 Department of Electrical and Computer Engineering, University of Delaware, 140 Evans Hall, Newark, DE 19716, USA; E-Mail: xinma@udel.edu; 3 Department of Materials Science and Engineering, University of Delaware, 201 DuPont Hall, Newark, DE 19716, USA; E-Mails: chelsera@udel.edu (C.R.H.); doty@udel.edu (M.F.D.); 4 Département de Génie Électrique, École de Technologie Supérieure, 1100 rue Notre-Dame Ouest, Montréal, QC H3C 0J9, Canada; E-Mail: luis-felipe.gerlein-reyes.1@etsmtl.net

**Keywords:** quantum dots, PbS, lead chalcogenide, surface ligand, EDT, 1,3-BDT, MPA, ammonium sulfide, photo-luminescence

## Abstract

The engineering of quantum dot solids with low defect concentrations and efficient carrier transport through a ligand strategy is crucial to achieve efficient quantum dot (QD) optoelectronic devices. Here, we study the consequences of various surface ligand treatments on the light emission properties of PbS quantum dot films using 1,3-benzenedithiol (1,3-BDT), 1,2-ethanedithiol (EDT), mercaptocarboxylic acids (MPA) and ammonium sulfide ((NH_4_)_2_S). We first investigate the influence of different ligand treatments on the inter-dot separation, which mainly determines the conductivity of the QD films. Then, through a combination of photoluminescence and transient photoluminescence characterization, we demonstrate that the radiative and non-radiative recombination mechanisms in the quantum dot films depend critically on the length and chemical structure of the surface ligands.

## 1. Introduction

Lead-chalcogenide colloidal quantum dots (QD) possess a tunable energy bandgap, high luminescence quantum efficiencies, a large absorption cross-section and low-cost solution processability. Recently, the appeal of these characteristics was emphasized in optoelectronic applications, including quantum dot solar cells, infrared photo-detectors, gas sensors and high efficiency infrared light emitting devices [1,2,3,4].

The key to achieving better QD optoelectronic devices consists of the fabrication of QD solids with low defect concentrations and efficient carrier transport. This depends critically on the chemical structure and the length of the capping ligand surrounding and interconnecting the quantum dots. During the colloidal QD synthesis, surface and mid-gap defects can be readily introduced due to the incomplete surface passivation. The mid-gap defect states can significantly reduce the luminescence quantum efficiency, trap the photo-generated carriers and pin the Fermi level in QD-based devices [5,6,7,8,9,10]. Therefore, proper passivation of these surface states is of paramount importance to produce more efficient QD devices [5,11,12,13,14].

Due to the characteristic hopping mechanism that dominates the charge transport in the quantum dot solids, the choice of the surface ligand also dictates the spatial separation between quantum dots and the tunneling barrier height for carriers. In other words, the choice of the surface ligand and its length play a crucial role with regards to the mobility and the conductivity of the quantum dot films. In general, bulky aliphatic ligands (with 8–18 carbons), such as trioctylphosphine (TOP) and oleic acid (OA), are typically employed in the synthesis to prevent particle aggregation and impart solubility to quantum dots by giving them a hydrophilic or hydrophobic surface [15,16]. However, these long ligands create strong insulating barriers between quantum dots and impede efficient carrier transport.

Indeed, significant work has been devoted to the ligand exchange process in an attempt to reduce the defect density to achieve lower non-radiative recombination losses and to minimize the interparticle spacing to promote carrier transport. In their pioneering work on QD solids, Murray *et al.* completely removed the oleic acid on PbSe QDs with diluted hydrazine solution to fabricate nanocrystal thin-film field-effect transistors with high conductivities [17]. Meanwhile, Moreels *et al.* demonstrated that lead-chalcogenide colloidal QDs were composed of a stoichiometric core surrounded by a Pb-rich surface [18], showing that ligands with high affinity to the lead ions can be used to effectively passivate the surface defects. Then, the short thiol ligand ethanedithiol (EDT) was employed for nanocrystal cross-liking into films and received significant attention [19,20,21]. Indeed, great advances have been achieved in photovoltaic and light-emitting devices based on the thiol-passivated PbS QDs. More recently, Tang *et al.* have reported on the passivation of quantum dots using atomic ligands, while Zhang *et al.* have explored the inorganically-interconnected nanocrystals [2,22].

While most of these reports focus on the chemical and electrical properties of the QD solids [23,24,25], the influence of surface ligand treatments on the light emission properties and the transient photoluminescence properties of the QD films has been relatively less well explored. In this paper, the influence of 1,2-ethanedithiol (EDT), 1,3-benzenedithiol (1,3-BDT), mercaptocarboxylic acids (MPA) and ammonium sulfide ((NH_4_)_2_S) treatments on the optical properties of QD films is examined. Among these ligands, Choi *et al*. have studied the time-photoluminescence and exciton dissociation mechanisms in EDT- and 1,3-BDT-treated QD films [26]. However, the effects of MPA treatments on the luminescence properties of the QDs are far less reported, even though it has been frequently employed for ligand exchange to achieve high efficiency QD solar cells [25,26]. On the other hand, the (NH_4_)_2_S treatment differs significantly from the other ligand treatments by creating inorganically-interconnected quantum dot solids through metal-sulfide bonds, while retaining quantum confinement properties. As such, we systematically studied and compared the effects of different surface ligand treatments on the photoluminescence properties and exciton recombination dynamics of the QD solids. In the future, we believe this will prove an important pathway to controllably engineer the properties of these QD-based films.

## 2. Results and Discussion

**Figure 1:**
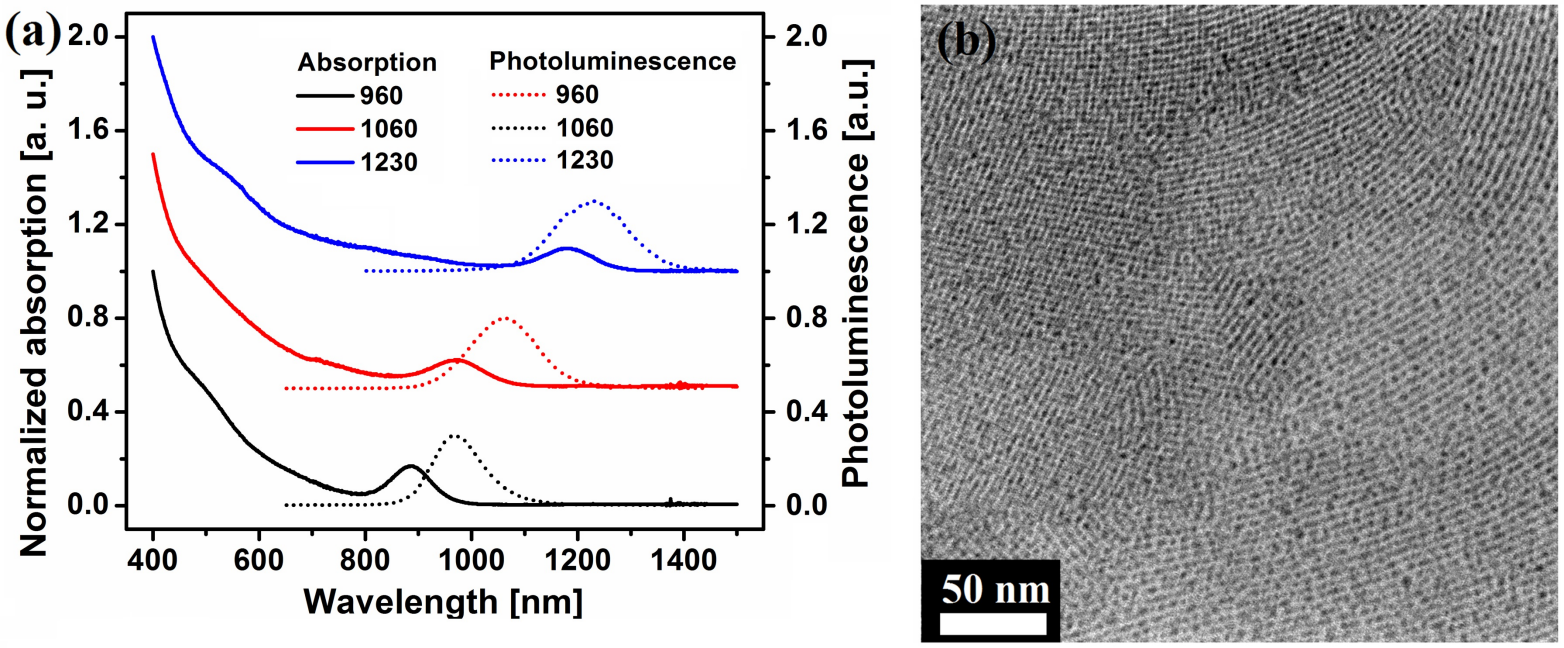
(**a**) Absorption (solid line) and photoluminescence (dotted line) spectrum of the as-synthesized PbS quantum dot (QD) with three different sizes; the QDs are identified using their photoluminescence peaks. “960” represents the QDs with photoluminescence peak at 960 nm; “1060” represents the QDs with photoluminescence peak at 1060 nm; “1230” represents the QDs with photoluminescence peak at 1230 nm. (**b**) TEM image of synthesized PbS QDs with an average size of ~3 nm.

The absorption (solid line) and photoluminescence (dotted line) spectrum of the as-synthesized PbS dots with different sizes are shown in Figure 1a. A Transmission Electron Microscopy (TEM) image of the PbS QDs with an average size of ~3 nm is shown in Figure 1b. The average size was extracted from the image using the software ImageJ. Previous work has demonstrated that EDT, 1,3-BDT, MPA and (NH_4_)_2_S treatments can effectively remove the oleic acid on QDs [22,26,27,28]. Here, we first investigate the effects of different surface ligand treatments on the inter-dot separation through TEM and High-Resolution Transmission Electron Microscopy (HRTEM).

**Figure 2:**
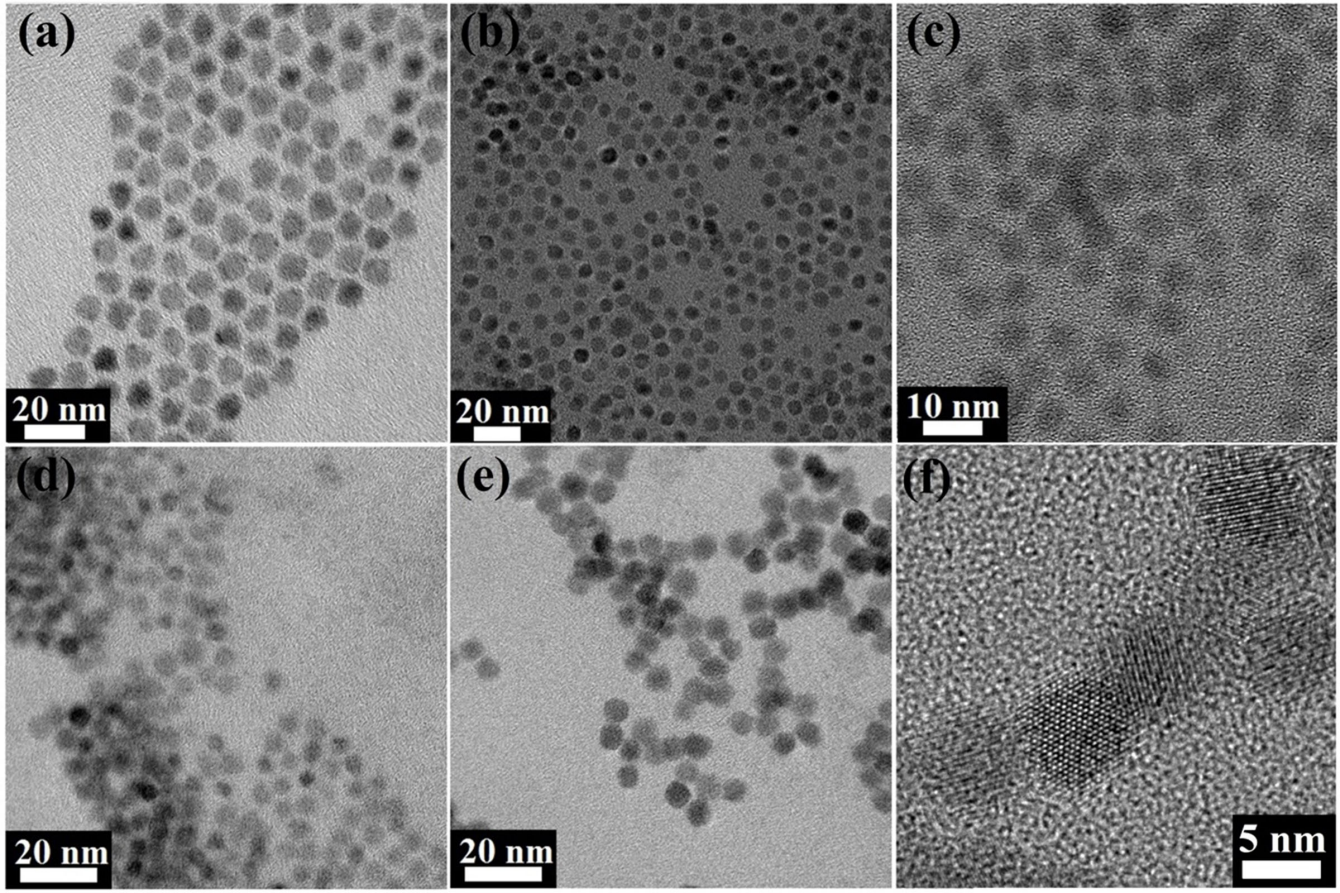
TEM and HRTEM images of (**a**) as-synthesized PbS QDs capped with oleic acid, with an interparticle distance of 10.2±0.8 nm; (**b**) PbS QDs after 1,3-BDT treatment, with an interparticle distance of 9.3 ± 0.8 nm; (**c**) PbS QDs after EDT treatment, with an interparticle distance of 7.8 ± 0.8 nm; (**d**) PbS QDs after MPA treatment, with an interparticle distance of 7.6 ± 0.8 nm; (**e**) PbS QDs after (NH_4_)_2_S treatment, with an interparticle distance of 6.7 ± 0.8 nm; (**f**) high-resolution TEM images of the (NH_4_)_2_S-treated PbS QDs.

Figure 2 shows the TEM images of the synthesized PbS QDs before and after cross-linking with 1,3-BDT, EDT, MPA and (NH_4_)_2_S. Among these, EDT is a dithiol molecule with a short C_2_ backbone chain; 1,3-BDT is a conjugated dithiol molecule with two thiol ligands arranged in a meta configuration on the benzene ring. It is slightly different from the 1,4-BDT, where the two thiol ligands are on the opposite side of the benzene ring; MPA has a C_2_ backbone chain with a thiol ligand at one end and a carboxylate acid on the other side [21,27,29]. As such, different surface ligand treatments result in drastically different inter-dot distances. To clearly resolve the inter-dot separation, relatively big PbS QDs having an average diameter of ~7.7 nm are chosen, and the center-to-center distance between QDs is measured based on the TEM images using the analysis software ImageJ.

As shown in Figure 2, while both EDT and 1,3-BDT are dithiol molecules, the EDT-treatment brings the QDs much closer than the 1,3-BDT-treatment does. On average, the measured interparticle distance of EDT-treated sample is 7.8 ± 0.8 nm, while that of the 1,3-BDT-treated sample is 9.3 ± 0.8 nm. This is because the shorter C_2_ backbone chain EDT molecule is less bulky than the aromatic 1,3-BDT. As shown in Figure 2c, it is likely that the two thiols on each side of the EDT molecule can directly bridge the two neighboring QDs. In contrast, the MPA molecule can bind to the QD surface in a bidentate fashion, since both the carboxylate group and the thiol ligand can coordinate to the lead ions [25]. Since both EDT and MPA have the C_2_ chain, the QDs treated with MPA shows an average inter-dot separation of 7.6 ± 0.8 nm, which is slightly smaller than that of EDT-treated sample. This suggest that MPA can also bind tightly to the QD surface.

Conversely, (NH_4_)_2_S treatment affects the QD film in a drastically different manner [22]. The chemical reaction triggered by the (NH_4_)_2_S in the QD exterior not only removes the original surface ligand, but also changes the QD composition. The reaction induced by the ligand treatment can be described by:

*Pb*(*OA*)_2_ + (*NH*_4_)_2_*S → PbS* + 2(*NH*_4_)*OA* (1)

The reaction presented in Equation (1) describes the conversion of the metal-surfactant interface into a sulfide-rich layer with metal-sulfide bond. The resultant QDs are bare and lack the surface encapsulation; thus, they can merge with the neighboring QDs by forming an interparticle metal-sulfide bond. As shown in the TEM image in Figure 2f, the neighboring QDs fuse with each other at the boundary. The resultant QD films possess very small interparticle spacing of 6.7 ± 0.7 nm, which is even smaller than the original size of the nanocrystals before treatment. This confirms the physico-chemical reaction induced by the treatment. In summary, if we compare the QD films after the different treatments with the OA-capped QD films in Figure 2a, the 1,3-BDT treatment can reduce the inter-dot separation by 0.9 nm; EDT treatment can reduce the inter-dot separation by 2.4 nm; MPA treatment can reduce the inter-dot separation by 2.6 nm; and (NH_4_)_2_S treatment can reduce the inter-dot separation by 3.5 nm.

### 2.1. Effects of the Surface Treatments on the Emission Properties of the QDs Solids

**Figure 3:**
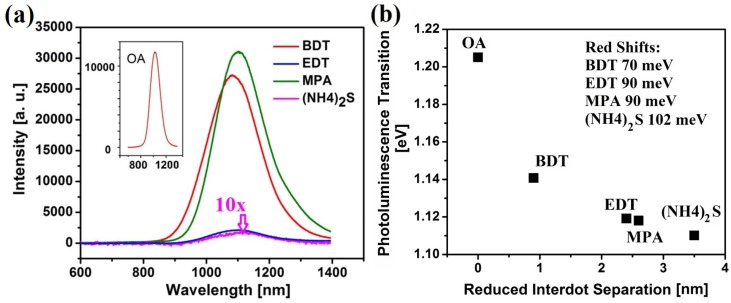
(**a**) The photoluminescence (PL) spectrum of one layer of spin-coated PbS QD films treated with different surface ligands. The inset shows the PL spectrum of the original oleic acid (OA)-capped QD films for reference only. (**b**) The photoluminescence peak transition *versus* the reduced inter-dot separations induced by different surface ligand treatments.

Removing the strongly-insulating oleic acid ligand from the QDs surface is known to trigger the Mott-type insulator to conductor transition [30], but it can also have a significant influence over the optical properties of the QD solids [14,19,21,27,31,32]. The photoluminescence characterization of the QD solids that were treated with different surface ligands is shown in Figure 3.

Pronounced red-shift in the QD photoluminescence peaks is observed. This effect can mainly stem from four possible causes [21,23,27,32]: (i) an increase of the physical size of the QDs; however, this can be easily ruled out, since the TEM images confirm that the size of the QDs remains the same after the surface treatment; (ii) a stronger electronic coupling among the neighboring QDs via the extension of the electron wavefunction outside the QDs, which reduces the quantum confinement; (iii) a resonant exciton energy transfer across the QD size distribution via a dipole-dipole interaction, where exciton flows from the wider band-gap QDs to the smaller band-gap QDs; however, this energy transfer process will result in an asymmetric emission line shape by quenching the photoluminescence (PL) on the high energy side, while enhancing the PL on the low energy side, which is inconsistent with the symmetric Gaussian emission peak observed in Figure 3; and (iv) charge transfer between the QDs and the surface ligands that delocalizes the electron wavefunction within the QD and relaxes the quantum confinement effect. This is also unlikely to be the main cause here. In such a case, one would expect the similar extent of red-shifts in both 1,3-BDT- and EDT-treated QD films, since either molecule has two thiol ligands that can bind to the lead ions on the QD surfaces. In addition, Choi *et al*. have demonstrated that the charge transfer from QDs to the surface ligand is not a prominent process in dithiol-treated PbS QD films [27]. Therefore, we can assess that the stronger electron coupling is the main factor contributing to the red-shift observed in the emission from the treated QD solids.

Indeed, the electron coupling energy *β* in QD films increases exponentially with a decreasing inter-particle distance. This coupling energy can be expressed as *β* ≈ *h*Γ, where Γ is the tunneling rate between neighboring QDs, which can be approximated as:

Γ ≈ *exp*[−2(2*m*^∗^∆*E/ћ*^2^)^1/2^∆*x*] (2)

where *m*^∗^ is the carrier effective mass, ∆*E* is the height of tunneling barrier and ∆*x* is the inter-dot distance between QDs [27]. From Equation (2), it can be seen that as the surface ligand treatments reduce the inter-dot separations, stronger electron coupling occurs. This extension of the carrier wavefunction in the QD film will alleviate the quantum confinement and red-shift the photoluminescence energies in the QD films. Figure 3b compares the emission peak energy after the different treatments with the 1.205 eV measured in the OA-encapsulated QD film. This result shows that the 1,3-BDT treatment induces an emission red-shift of 70 meV, while the less bulky two-carbon EDT and MPA ligands induce a larger red-shift of 90 meV. In contrast, the interconnected (NH_4_)_2_S-treated QD- films display an even more significant red-shift of 102 meV. This further proves that the shorter inter-dot separations can enhance the electron coupling, thereby reducing the quantum confinement and giving rise to a greater red-shift in QD photoluminescence.

In addition, the surface ligand treatments induce photoluminescence intensity changes. The photoluminescence intensity of the 1,3-BDT-, EDT-, MPA- and (NH_4_)_2_S-treated QD films are compared in Figure 3a. It should be noted that the photoluminescence of the OA-capped QD film is not compared here, since the surface ligand treatment will change the dielectric constant of the QD films and enhance the QD absorption [21,27]. On the other hand, based on our previous work and that of [1,25,27], we speculate that the absorption of the 1,3-BDT, EDT and MPA would have only small differences. The assembly of conducting QD solids typically leads to significant photoluminescence quenching due to exciton dissociation in strongly-coupled QD solids and the transfer of carriers to non-radiative sites [24,27,33]. Indeed, a low PL intensity can be observed after the EDT treatment in Figure 3a, which can be explained by two phenomena. First, the short EDT molecule results in a tightly-packed and highly-coupled QD solid that induces a high exciton dissociation rate [27]. Moreover, EDT is volatile, and it will evaporate after short exposure to air, leaving the QDs in the solid unpassivated. In our previous work, we have established that solids treated with EDT result in significantly higher defect concentrations compared with 1,3-BDT [28]. Meanwhile, Sargent *et al.* have demonstrated that the EDT treatment yields more than a three-times higher defect concentration compared with MPA [25]. This leads to the formation of deep mid-bandgap non-radiative recombination centers that significantly quench the luminescence.

In comparison, the 1,3-BDT-treated QD solids exhibit a dramatically higher PL (integrated intensity), because the conjugated 1,3-BDT molecule is much more stable in air thanks to a better QD passivation. In addition, the longer inter-dot distance in 1,3-BDT-treated films gives rise to much smaller exciton dissociation rates.

For the MPA ligand, the existence of two types of functional groups leads to QD passivation of better chemical diversity and to an enhanced stability in air. This makes perfect sense, since it was reported that MPA can passivate different types of surface states, resulting in low densities of shallow trap states in QD films [6,25]. Indeed, MPA treatment results in even higher PL emission intensities. In contrast, the (NH_4_)_2_S treatment connects the neighboring QDs with metal sulfide bonds, while leaving the QD surface unpassivated. While the treated QD solid retains strong quantum confinement, as evidenced by the position of the PL peak, the lack of surface passivation leads to high densities of surface defects that drastically quench the PL intensity. As such, the films treated with (NH_4_)_2_S display more than ten-times weaker PL intensity compared with the EDT-treated films, shown in Figure 3a.

To better understand the PL quenching mechanisms associated with those observations, we turned to time-resolved PL spectroscopy. In lead-chalcogenide quantum dots, the radiative recombination of excitons has relatively long lifetimes ranging from hundreds of nanosecond to a few microseconds [27,34].

**Figure 4:**
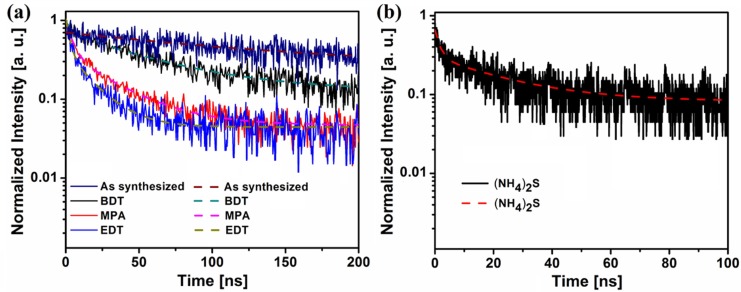
(**a**) Time-resolved PL spectrum of the spin-cast QD film. Dark blue trace, as-synthesized oleic acid-capped film; black trace, 1,3-BDT-treated QD film; red trace, MPA-treated QD film; blue trace, EDT-treated QD film. (**b**) The (NH_4_)_2_-treated QD film. The dotted red line shows the bi-exponential fitting curve.

For oleic acid-capped QD films, a long exciton lifetime of 142 ns with single exponential decay can be observed, as shown in Figure 4a. As one can see in Figure 4, the surface ligand treatments significantly reduce the exciton lifetime to several tens of nanoseconds and result in a multi-exponential decay feature, suggesting that the treatments significantly change the de-excitation mechanisms in QD films. After treatment, the transient photoluminescence decay can be fitted with a bi-exponential curve, presented in Equation (3):

(3) $$I\left(t\right)=A_{1}\cdot exp\left(-\frac{t}{T_{1}}\right)+A_{2}\cdot exp\left(-\frac{t}{T_{2}}\right)+A_{0}$$

where *T*_1_ represents the fast-decay component and *T*_2_ represents the slow-decay component, while *A*_1_ and *A*_2_ correspond to their amplitudes. Meanwhile, the intensity-averages reduced exciton lifetime *T*_*r*_ can be calculated using:

(4) $$T_{r}=\frac{A_{1}T_{1}^{2}+A_{2}T_{2}^{2}}{A_{1}T_{1}+A_{2}T_{2}}$$

The shortened exciton lifetime is likely to be caused by the creation of non-radiative recombination pathways via the surface ligand treatments, including the exciton dissociation via charge transfer, the resonant energy transfer and the non-radiative recombination via bulk defect states and mostly through surface states [28]. The charge transfer process depends exponentially on the inter-dot separation, while the resonant energy transfer depends on the inter-dot separation *R* proportionally to 1/*R*^6^.

For the as-synthesized QD films, the oleic acid can provide decent surface passivation to the quantum dot while the long ligand will prevent efficient charge transfer and prohibit any resonant energy transfer. This is why we can see the slow radiative recombination process dominating the oleic acid-encapsulated QD films’ emission. After the surface ligand treatments, the inter-dot separation is significantly reduced, and the non-radiative recombination becomes prominent. Indeed, the 1,3-BDT-treated QD film exhibits a much-shortened exciton lifetime of ~54 ns. In contrast, the EDT-treated QD film has the shortest reduced exciton lifetime of ~17 ns. This is due to the fact that the EDT-treatment not only results in short inter-dot separation that facilitates exciton dissociation, but also creates high densities of surface states that induce rapid non-radiative recombination [31].

In contrast, the MPA-treated film shows a medium exciton lifetime of ~36 ns, which is highly reasonable. Indeed, the MPA treatment results in similar inter-dot separation compared to the EDT treatment. However, MPA provides a much more efficient surface passivation of the surface and defect states compared with EDT. Therefore, the reduced exciton lifetime is significantly longer than that of the EDT-treated sample, while shorter than that of the 1,3-BDT-treated sample.

Finally, the PL decay and fitting curves of the (NH_4_)_2_S-treated film are shown in Figure 4b. Owing to the weak emission of the sample and the sensitivity limit of the silicon detector, the signal-to-noise ratio of the (NH_4_)_2_S is worse than the other samples. However, a shortest fast decay constant *T*_1_ of 1.6 ns can be extracted from the fitting. Considering the almost inexistent inter-dot separation and the unpassivated QD surface, this fast and strong non-radiative recombination should be expected for the (NH_4_)_2_S-treated sample. In Table 1, a summary of the fitting parameters extracted from the time-resolved photoluminescence measures is presented.

**Table 1:** Transient photoluminescence fitting results and the calculated intensity reduced exciton lifetime *T*_*r*_.

Ligand Treatment	*T*_1_ (ns)	*A*_1_	*T*_2_ (ns)	*A*_2_	*T*_*r*_ (ns)
As synthesized	142 ± 10	0.23	142 ± 10	0.23	141.7
1,3-BDT	4.3 ± 1.1	0.26	56 ± 4	0.58	54
EDT	2.5 ± 0.2	0.64	17 ± 1	0.36	14
MPA	4.3 ± 0.2	0.6	31.2 ± 1.4	0.34	24
NH_4_)_2_S	1.6 ± 0.14	0.35	23 ± 1.1	0.21	19.4

## 3. Experimental Section

The nanocrystal synthesis is done in a nitrogen-purged glove-box. The PbS QDs are synthesized following the method reported by Hines and Scholes [35]. Lead oxide (2 mmol), octadecene (ODE, 10 mL) and oleic acid (OA, 4 mmol) are first added to a single three-neck flask with a heating mantle. The mixture is then heated at 120 °C until all of the lead oxide (lead acetate) dissolves under continuous stirring. The mixture is then kept at the desired temperature between 70 to 150 °C, which plays a role in the QD average size depending on the device’s needs. Subsequently, 5 mL of hexamethyldisilathiane solution (1 mmol) in ODE is quickly injected into the reaction flask under vigorous stirring. The color of the reaction solution rapidly changes from dark blue to red and, finally, to black. The reaction solution is annealed for 5 min at the injection temperature, then the heating mantle is removed, and the solution reaction is quenched by placing the flask under cool water.

The QDs are precipitated by injecting excessive amounts of acetone into the reaction solution and then centrifuging. The precipitates are dried in vacuum and re-dispersed in hexane. To ensure adequate removal of the reaction residues, precipitation and re-dispersion are repeated at least two times. The QD solution is finally filtered with 200-nm polytetrafluoroethylene filters. The size of the QDs can be controlled by adjusting the injection temperature and, especially, the amount of oleic acid used in the reaction.

To perform the surface ligand treatment, the QD solution with an approximate concentration of 15 mg/mL is spin-coated on a pre-cleaned glass substrate at 2,000 rpm. Once dried, the substrate is dipped into the surface ligand solution for 5 or 10 s (5 s for 0.02 M EDT in anhydrous acetonitrile, 0.02 M 1,3-BDT in anhydrous acetonitrile and 10% by volume of MPA in methanol, compared with 10 s for 0.001 M (NH_4_)_2_S in methanol), then rapidly removed and allowed to dry. The thickness of the QD film can be increased by repeating the deposition cycles multiple times. As a result, shiny QD films with areas up to a few square centimeters are fabricated using this process.

TEM images are acquired with JEM-2010F (JEOL, Tokyo, Japan) under an accelerating voltage of 200 keV. To prepare the samples for inter-dot separation analysis, a low concentration of QDs, approximately 1 mg/mL, is spin-coated on a glass substrate covered with a thin layer of poly(3,4-ethylenedioxythiophene) polystyrene sulfonate (PEDOT:PSS). After the QD surface ligand treatment, the sample is immersed into deionized water dissolving this PEDOT:PSS layer. The QD film peels off from the substrate and then is transferred to a TEM grid for characterization after it dries.

The photoluminescence (PL) measurements are performed at room temperature using a Jobin-Yvon iHR320 triple-grating spectrometer (Horiba Scientific, Kyoto, Japan) equipped with a Symphony thermoelectrically-cooled InGaAs detector array. For PL excitation, we used a 250-mW frequency-stabilized TORUS laser (Laser Quantum, Cheshire, UK) operating at 532 nm. For time-resolved PL measurements, we use the Mira-900 femtosecond pulsed laser (Coherent, Santa Clara, CA, USA) that runs at a repetition rate of 151 kHz. The excitation wavelength is 780 nm and is filtered from the PL with an 800 nm-long pass filter and an ND1 neutral density filter. The time-resolved PL is detected using a Perkin-Elmer avalanche photodiode with a PicoHarp time-correlated single photon counting system (TCSPC), and the spectral bandwidth is 25 nm.

## 4. Conclusions

In conclusion, we studied the effects of different surface ligand treatments on the inter-dot separation and the recombination mechanisms of nanocrystalline PbS quantum dot films. Shorter inter-particle separation and a higher density of surface states in the EDT-treated structures result in significant quenching of the emission via surface and defect state-assisted non-radiative recombination. In contrast, the 1,3-BDT treatment can moderately reduce the inter-dot separation, while efficiently passivating the QD surfaces, leading to high QD film emission. Meanwhile, the short MPA ligand can also shorten the inter-dot separation, while enhancing the QD passivation, due to better chemical diversity, thereby significantly increasing the QD emission compared to EDT-treated QD films. Finally, the (NH_4_)_2_S treatment can interconnect the neighboring quantum dots by forming metal-surfactant bonds at the QD boundaries. While the quantum confinement is preserved, the exchanged ligand completely removes the capping agent and leaves the QD bare and unpassivated, leading to a dramatic quenching and the shortest photoluminescence decay time constant of ~1.6 ns due to strong non-radiative recombination. The results are summarized in Table 1.
